# Preventing child marriages: first international day of the girl child “my life, my right, end child marriage”

**DOI:** 10.1186/1742-4755-9-31

**Published:** 2012-11-20

**Authors:** Joar Svanemyr, Venkatraman Chandra-Mouli, Charlotte Sigurdson Christiansen, Michael Mbizvo

**Affiliations:** 1 Department of Reproductive Health and Research, World Health Organization, 20 Avenue Appia, 1211, Geneva 27, Switzerland; 2 Adolescent Sexual and Reproductive Health, Department of Reproductive Health and Research, World Health Organization, 20 Avenue Appia, 1211, Geneva 27, Switzerland; 3 Department of Maternal, Newborn, Child and Adolescent Health, World Health Organization, 20 Avenue Appia, 1211, Geneva 27, Switzerland; 4 Department of Reproductive Health and Research, World Health Organization, 20 Avenue Appia, 1211, Geneva 27, Switzerland

## Abstract

On 17 November 2011, the United Nations General Assembly adopted a resolution (A/RES/66/170) designating 11 October as the first International Day of the Girl Child choosing ending child marriages as the theme of the day. Child marriage is a fundamental human rights violation and impacts all aspects of a girl’s life. These marriages deny a girl of her childhood, disrupts her education, limits her opportunities, increases her risk of violence and abuse, and jeopardizes her health. The article presents data about the prevalence and effects, contributing factors and recommends action for prevention.

## Background

Child marriage, defined as a formal marriage or informal union before age 18, is a reality for both boys and girls; however girls are disproportionately the most affected. Globally nearly one in three girls are married before the age of 18, and one in seven is married before the age of 15. An estimated 10 million child marriages occur every year [1].

The extent of early marriage varies between countries and regions: the highest rates are found in West Africa, followed by southern Asia, northern Africa/the Middle East and Latin America. However, given southern Asia’s population size and rates of early marriage, approximately half of girls in early marriages live there. There are also big differences in prevalence among countries in the same region, with high rates in poorer and less developed parts as well as in rural areas [1,2].

While data from 47 countries show that, overall, the median age at first marriage is gradually increasing, this improvement has been limited primarily to girls of families with higher incomes. Unfortunately, the pace of change remains slow. While 48 per cent of women 45–49 years old were married before the age of 18, the proportion has only dropped to 35 per cent of women 20–24 years old [2].

The effects and consequences are vast - early marriage leads to early pregnancy which increases the risk of complications during pregnancy and childbirth. The increased risk of death or serious lasting complications such as obstetric fistula is greater for girls in early and middle adolescence [3].

Maternal deaths related to pregnancy and childbirth are an important component of mortality for girls aged 15–19 worldwide, accounting for 70,000 deaths each year. The risk of dying from pregnancy-related causes is 4 times higher for adolescents under 16 years than for women in their early twenties. [4]. If a mother is under the age of 18, her infant’s risk of dying in its first year of life is 60 per cent greater than that of an infant born to a mother older than 19. Infants born to young mothers are more likely to be born underweight, premature and to experience serious health problems [5]. Child marriage has also been shown to increase the likelihood of HIV infection and of domestic violence [6,7]. Further, child brides are at risk of violence, abuse and exploitation [8].

Early marriage perpetuates the cycle of illiteracy and poverty. Around the world, more girls are enrolled in school than ever before. These girls are much less likely to be married at an early age, however, sadly, school enrolment drops sharply after five or six years of schooling [9]. Child marriage often results in girls leaving school, reducing their opportunity to learn and to gain skills that would enable them to start an income generating activity or to find a job. It thereby increases the likelihood of low levels of education and employment [10]. Importantly child marriage often results in separation from family and social networks. It reduces the girls’ possibility to obtain practical and emotional support and to participate in community activities with important consequences for their sense of wellbeing [11].

Poverty is correlated with higher rates of child marriage. Poor families may not have the resources to keep girls in school or even to feed and clothe them. Because of prevailing gender norms they may prefer to use the limited resources they have for their sons rather than their daughters. Poor families may see marriage as a means of reducing their economic burden- in some places, economic gains such as a bride price (dowry) provide them with an incentive to marry their daughters early [12-15]. In some parts of the world, girls are expected to marry and start having children in early adolescence. Where prevalent child marriage functions as a social norm; parents feel pressured by existing expectations and traditions or by economic hardships. Families may marry their daughters at a young age because they believe that marriage will protect their daughters from premarital sex and pregnancy or because of concerns that if their daughters are not married early according to local traditions, they may not be married at all [12-15].

There is an ongoing effort to pass national laws declaring the minimum age of marriage in many countries, a strategy that should be commended. Where laws are in place, efforts are needed to ensure that they are followed.

Child marriage can be prevented. Marrying girls under 18 years old is rooted in gender discrimination. Delaying the age of marriage requires working with communities to question, challenge and change such norms. An empowered and informed girl needs a favorable family and social environment to attain her maximum potential. Actions are needed to influence family and community norms related to delayed marriage [16].

Representatives from thirty countries at the 65th Assembly of the World Health Organization (May 2012) noted in their contributions to the discussions on "Early marriages, adolescent and young pregnancies” that early marriage is illegal in most places where it occurs; it is a violation of the rights of girls, and it has detrimental health and social consequences on adolescent girls, their families and communities [17].

The Commission on Population and Development (CPD) stated in its 2012 resolution that it is “*Concerned* that early and forced marriage and forced sexual relationships have adverse physical, social and psychological effects on adolescents and young girls and violate their human rights, and that early childbearing and early and forced marriage reduce opportunities for adolescent and young girls to complete their education, develop employable skills and participate in community development.”

Furthermore, the CPD resolution **“***Urges* Member States to enact and strictly enforce laws to ensure that marriage is entered into only with the free and full consent of the intending spouses and to enact and strictly enforce laws concerning the minimum legal age of consent and the minimum age for marriage, and to raise the minimum age for marriage, where necessary” [18].

There is a growing groundswell of action. One example of this is Every Woman Every Child, the Global Strategy for Women’s and Children’s Health, launched by the Secretary General of the United Nations. This global initiative aims to prevent maternal and childhood mortality, and includes activities to prevent child marriage as part of efforts to prevent adolescent pregnancy and pregnancy-related mortality and morbidity [19].

Furthermore the evolving movement of Girls Not Brides (http://www.girlsnotbrides.org), a global partnership of over 180 organizations, is working to end child marriage by raising awareness of the scale and impact of the problem and mobilizing the support needed to end it. Its members also support the victims of child marriage. Created in 2010 by The Elders, an independent group of eminent global leaders brought together by Nelson Mandela in 2007 (http://www.theElders.org) Girls Not Brides has rapidly grown into a powerful force for change.

In 2011 WHO published guidelines on “Preventing early pregnancy and poor reproductive outcomes among adolescents in developing countries” [20]. Among the six set of recommendations, one relates to the prevention of marriage before the age of 18. The guidelines present direction regarding what is needed to be done to prevent early marriage:

•Prohibit early marriage – a call for policy makers to put in place and enforce laws that prohibit marriage before the age of 18.

•Keep girls in school – a need to increase education opportunities for girls, as this has positive effects on their health, and also decreases the chance of getting married.

•Influence cultural norms that support early marriage – a need to work with all stakeholders to challenge and change norms around early marriage.

The guidelines also highlighted areas in which evidence needs to be strengthened:

•Types of interventions that can result in the formulation of laws and policies to protect adolescents from early marriage (e.g. public advocacy).

•A better understanding of how economic incentives and livelihood programmes can work to delay the age of marriage among adolescents.

•Better methods to assess the impact of education and school enrolment on the age of marriage.

•Assess existing interventions to inform and empower adolescent girls, their families and their communities to delay the age of marriage, and the scalability of such projects.

Working in conjunction with agencies within and outside the United Nations system, WHO is fully committed to supporting countries in applying the Guidelines’ recommendations and to strengthening the evidence base in this area. One of its priority areas of research is to “Understand the determinants of early pregnancy and identify effective and feasible legal, social and economic measures of preventing it” [21].

This new initiative aims to promote understanding, engagement and accountability of all actors in this area. It seeks to fulfill the rights of girls and young women to grow and develop to their full potential, and the opportunity to decide when and with whom they want to become pregnant.

## Competing interests

We do not have political, personal, religious, academic, ideological, intellectual, commercial or any other competing interest.

## Authors’ contributions

JS and VCM drafted the manuscript and prepared the final version, CSC participated in literature review and commented the draft. MB participated in commenting the draft. All authors approved the final manuscript.
